# Structure–property–degradability relationships of varisized lignocellulosic biomass induced by ball milling on enzymatic hydrolysis and alcoholysis

**DOI:** 10.1186/s13068-022-02133-x

**Published:** 2022-04-04

**Authors:** Xueli Chen, Dingping He, Tao Hou, Minsheng Lu, Nathan S. Mosier, Lujia Han, Weihua Xiao

**Affiliations:** 1 Engineering Laboratory for AgroBiomass Recycling & Valorizing, College of Engineering, China Agricultural University (East Campus), 17 Qing-Hua-Dong-Lu, Haidian district, P.O. Box 191, Beijing, 100083 China; 2 Laboratory of Renewable Resources Engineering (LORRE), Purdue University, West Lafayette, IN 47907 USA; 3 Department of Agricultural and Biological Engineering, Purdue University, West Lafayette, IN 47907 USA; 4 School of Light Industry and Food Engineering, Guangxi Key Laboratory of Clean Pulp and Papermaking and Pollution Control, Guangxi University, Nanning, 530004 China

**Keywords:** Lignocellulose, Ball milling, Size reduction, Enzyme hydrolysis, Alcoholysis

## Abstract

**Background:**

Valorization of lignocellulosic biomass to obtain clean fuels and high-value chemicals is attractive and essential for sustainable energy and chemical production, but the complex structure of biomass is recalcitrant to catalytic processing. This recalcitrance can be overcome by pretreating biomass into deconstructable components, which involves altering the structural complexities and physicochemical properties. However, the impact of these alterations on biomass deconstruction varies considerably, depending on the pretreatment and subsequent conversion type. Here, we systematically describe the changes in structure and properties of corn stover after ball milling as well as their influence on the following enzymatic saccharification and acid-catalyzed alcoholysis, with the aim of elucidating the relationships between structures, properties and deconstructable potential of lignocellulosic biomass.

**Results:**

Ball milling causes dramatic structural changes, since the resistant plant cell walls are destroyed with size reduction to a cellular scale, leading to the increase in surface area and reducing ends, and decrease in crystallinity and thermal stability. As a result, ball-milled corn stover is more susceptible to enzymatic saccharification to fermentable sugars and provides more industrially viable processing approaches, as it is effective at high solids loading and minor enzyme loading, without any other pretreatment. Acid-catalyzed alcoholysis of corn stover to biofuels, on the other hand, is also enhanced by ball milling, but additional processing parameters should be tailored to the needs of efficient conversion. Further, a detailed examination of process variables coupled with a kinetic study indicates that acid-catalyzed alcoholysis is limited by the process variables rather than by the substrate parameters, whereas ball milling facilitates this reaction to some extent, especially under mild conditions, by lowering the activation energy of corn stover decomposition.

**Conclusions:**

The efficient catalytic conversion of biomass is closely related to its structure and properties, an understanding of which offers prospects for the rational improvement of methods aimed at more economic commercial biorefineries.

**Supplementary Information:**

The online version contains supplementary material available at 10.1186/s13068-022-02133-x.

## Background

Lignocellulosic biomass, the major component of agricultural and forestry waste, is a widely available, low-impact, and inexpensive feedstock to replace or minimize the carbon currently supplied by petroleum [1]. It has a complex composition mainly comprising cellulose, hemicellulose, and lignin, as well as small amounts of minerals and numerous extractives [2]. These renewable sources of carbon make lignocellulosic biomass an attractive candidate for the sustainable production of chemicals and fuels [3]. Such characteristics endow biomass with extraordinary potentials for future biorefinery conversion from technical, economic, and environmental perspectives. Yet, unlocking these potentials requires proper catalytic approaches to derive value from biomass components [4].

The biochemical transformation of lignocellulosic biomass with enzymes has been considered one of the most appropriate means for depolymerizing its polysaccharides into sugars, which are platform molecules for further transformation into diverse fuels and chemicals via microbial fermentation or chemical conversion [5]. The advantages of enzymatic catalysis include its highly specific and selective nature compared with other catalysts, low environmental impact, and milder reaction conditions. Conversely, its limitations primarily lie in low tolerance to changes in reaction conditions and the relatively high cost of enzyme production [6]. Instead, the thermochemical conversion of lignocellulosic biomass into value-added chemicals seems to be a simpler option, where inexpensive acids such as sulfuric acid are used as catalysts [7]. One attractive route is acid-catalyzed conversion of biomass into valuable platform chemicals including alkyl glucoside, 5-alkoxymethylfurfural, and alkyl levulinate in alcoholic media [8, 9]. Alcoholysis of lignocellulosic biomass has the advantages of facile product separation and minimum undesired byproduct formation relative to catalytic hydrolysis in an aqueous medium [10]. While these methods exhibit much promise for transforming lignocellulosic biomass into a wide array of attractive products, they face tremendous obstacles due to the structural complexity of biomass. Biomass is primarily constituted of aligned bundles of partially crystalline fibrils assembled in an amorphous matrix of hemicellulose and lignin [11]. This assembly of lignocellulosic biomass resists its deconstruction, hindering cost-effective conversion [12]. To overcome biomass recalcitrance, various pretreatment methods, involving physical, chemical, biological, and solvent processes, have been extensively studied and developed to produce an intermediate that is readily converted by enzymes or other catalysts [13].

As one of the simplest physical pretreatment methods, ball milling disrupts the structural barriers to lignocellulosic biomass conversion by fragmenting its macroscopic particles [14]. In addition to structural changes, fragmentation of biomass is always accompanied by modifications in its physicochemical properties, favoring further biological or chemical degradation [15, 16]. For instance, the glucose yield by the enzymatic hydrolysis of rice straw was found to be a function of its particle size and crystallinity, which are dramatically changed by the mechanical fragmentation [17]. A significant amount of literature is available regarding the characterization of structural features of lignocellulosic substrates after mechanical deconstruction and their contributions to enzymatic hydrolysis [15, 17–19]. Very few studies have focused on the benefits of mechanical deconstruction on enzymatic hydrolysis under industrially relevant conditions, such as low enzyme loadings and high solids concentrations [20]. Although experiments by Lu et al. [20] showed that ball milling is an efficient process for high-solids enzymatic hydrolysis with improved sugar yields, the dependence of these improvements on feedstock’s properties was not examined in detail.

Recent work by Liu et al. [21] also revealed that ultrafine grinding modifies corn stover and facilitates its acid-catalyzed alcoholysis to produce ethyl levulinate in multiple ways. It increases reaction accessibility because of increased surface area, reduces energy barrier to alcoholysis due to cellulose decrystallization, and leads to more reaction sites caused by partial depolymerization during grinding [21]. The influence of these changes on product distribution, however, is not known; nor has it been compared with that of the process variables to evaluate the extent of conversion enhancement.

Despite enhancing the reactivity of lignocellulose, ball milling is still energy-intensive and challenging to scale up [22, 23]. In addition, the effect of ball milling may differ between enzymatic hydrolysis and alcoholysis. In this respect, it is critical to balance the energy consumption and benefits of pretreatment, in which the enhancement in biomass degradability should be evaluated as a function of the changes in properties caused by the pretreatment. Therefore, a thorough understanding of the relationships among structures, properties, and degradability is necessary.

**Fig. 1:**
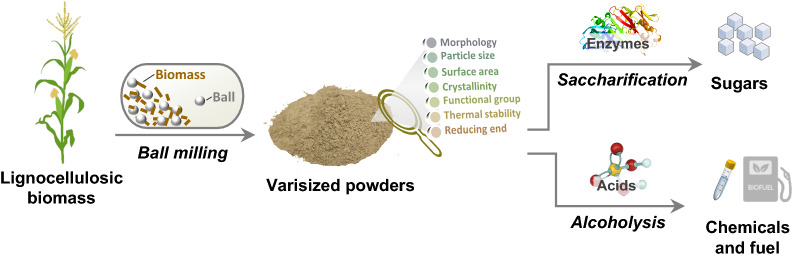
Schematic illustration for the preparation and characterization of varisized lignocellulosic biomass followed by enzymatic saccharification or catalytic alcoholysis

To address these needs, we herein detail the relationships among structure, properties and degradability as well as the determining factors for different biomass conversions (Fig. 1). Varisized corn stover samples were first prepared by ball milling, followed by a comprehensive characterization of their structure and properties, involving surface morphologies, particle size distribution, specific surface area, crystallinity, thermal stability, and reducing-end concentration. These corn stover samples were also subjected to enzymatic saccharification and acid-catalyzed alcoholysis, respectively, to evaluate the digestibility. Then, the relationships between digestibility and physicochemical properties of corn stover were determined through a correlation analysis. Additionally, we compared the relative contributions of substrate parameters and process variables to different conversions. Their effects on acid-catalyzed alcoholysis were further studied by systematically examining the process variables together with a kinetic study. Finally, we demonstrated the benefits of ball milling on enzymatic hydrolysis of corn stover under industrially relevant conditions.

## Results and discussion

### Changes in the physicochemical properties of corn stover with ball milling

**Fig. 2:**
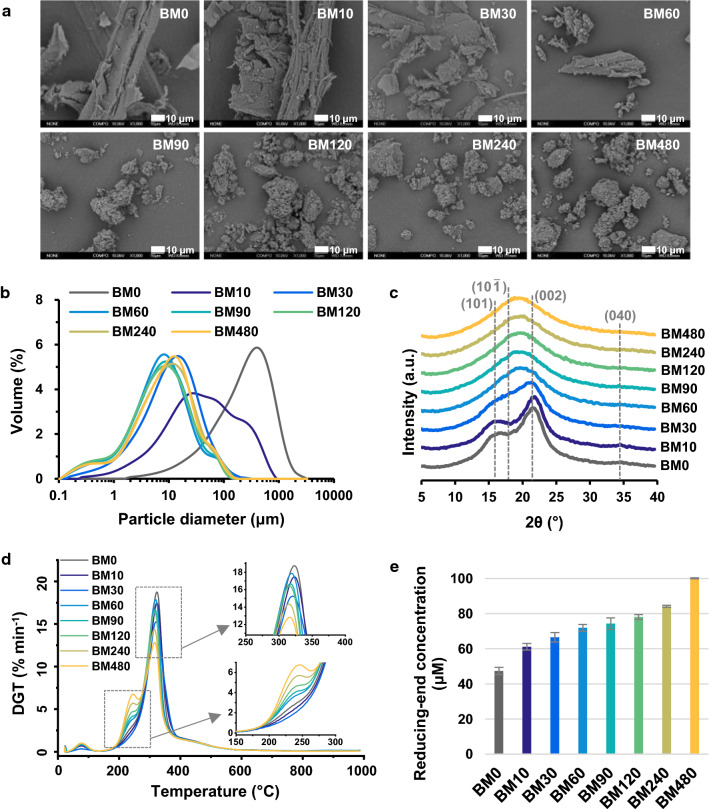
Characterization of corn stover samples after ball milling. **a** Scanning electron microscopy (SEM) images; **b** particle size distribution; **c** XRD patterns; **d** differential thermogravimetry (DGT) curves; and **e** reducing-end concentration of different corn stover samples

The morphology, particle size, and surface properties of biomass play a crucial role in its conversion [24], therefore such characteristics were investigated first. The SEM images in Fig. 2a show the changes in the surface morphology of corn stover after ball milling. Ball milling greatly alters the surface morphology of corn stover in addition to reducing the particle size. After a short time of ball milling (10 min), apparent cracks occurred on the surface of fibrous particles relative to the smooth surface of raw corn stover. When the ball milling lasted for 30 min, the corn stover lost its original fibrous shape and changed to small irregular particles. Longer ball milling time resulted in a large number of agglomerates with honeycomb structures. The resulting porous structure of corn stover can be confirmed by the increased specific surface areas from 1.12 to 4.07 m^2^ g^−1^ after ball milling for 60 min (Table 1). However, further extending ball milling time did not increase the specific surface area. Instead a slight reduction occurred at times from 60 to 480 min. This finding reveals that the effect of ball milling had reached the maximum at 60 min, because the corn stover powders tend to agglomerate with a much longer time [21]. The results agree with the changes in particle size. As observed in the SEM images, ball milling dramatically reduced the particle size of corn stover with median particle size (*D*_50_) changing from the original 263.00 μm to 39.13 and 7.18 μm after 10 and 60 min ball milling, respectively (Table 1). However, the particle size started to slightly rise after 60 min due to the agglomeration reported previously [17]. As shown in Fig. 2b, the particle size distribution of corn stover was also narrowed by the ball milling process, which is consistent with what is observed in the SEM. These observations demonstrate that the accessibility of biomass is enhanced by decreasing particle size and increasing specific surface area.

**Table 1:** Particle size distribution, specific surface area, and crystallinity of corn stover samples

Sample	*D*_10_ (μm)	*D*_50_ (μm)	*D*_90_ (μm)	Span	SSA (m^2^ g^−1^)	CrI (%)
BM0	38.17 ± 0.50^d^	263.00 ± 0.82^e^	828.33 ± 8.73^d^	3.00 ± 0.03^a^	1.12 ± 0.08^a^	40.68 ± 0.39^e^
BM10	4.98 ± 0.02^c^	39.13 ± 0.21^d^	276.00 ± 2.83^c^	6.93 ± 0.05^f^	2.30 ± 0.11^b^	36.03 ± 0.70^d^
BM30	1.92 ± 0.01^b^	11.33 ± 0.95^c^	42.27 ± 0.95^b^	3.56 ± 0.07^b^	3.35 ± 0.11^c^	20.69 ± 1.27^c^
BM60	1.20 ± 0.01^a^	7.18 ± 0.02^a^	30.10 ± 0.67^a^	4.03 ± 0.09^d^	4.07 ± 0.32^d^	14.06 ± 0.42^b^
BM90	1.16 ± 0.04^a^	7.39 ± 0.20^a^	32.37 ± 0.33^a^	4.22 ± 0.04^e^	4.01 ± 0.12^d^	9.74 ± 0.75^a^
BM120	1.06 ± 0.01^a^	7.42 ± 0.04^a^	32.80 ± 1.61^a^	4.28 ± 0.19^e^	4.01 ± 0.03^d^	9.34 ± 0.72^a^
BM240	1.32 ± 0.03^a^	8.90 ± 0.11^b^	36.40 ± 0.56^ab^	3.94 ± 0.01^ cd^	3.75 ± 0.23^ cd^	7.80 ± 0.45^a^
BM480	1.38 ± 0.02^a^	9.08 ± 0.11^b^	35.67 ± 1.24^ab^	3.77 ± 0.10^c^	3.62 ± 0.13^ cd^	7.45 ± 0.02^a^

Data are presented as mean ± standard deviation. Values in the same column with different letters are significantly different (*p* < 0.05)

In addition to particle size and morphology, crystallinity is also a key factor determining cellulose conversion and is affected by ball milling [25]. As depicted in Fig. 2c, the diffraction pattern for raw corn stover has three peaks at around 16°, 22° and 35°, corresponding to (101), (002), and (040) of crystalline cellulose, respectively [26]. These crystalline peaks became less intense after ball milling. Accordingly, the crystallinity index (CrI) of corn stover decreased from 40.68 to 20.69 after the 30 min ball milling pretreatment (Table 1). After ball milling for 60 min, the peaks indicative of crystalline phases almost completely disappeared and only a broad peak attributed to amorphous cellulose can be observed. This observation indicates that ball milling destroys the crystalline structure of cellulose in corn stover and forms amorphous cellulose, which is more accessible and degradable as previously reported [27]. Such structural destruction could reduce the thermal stability of corn stover by lowering the energy required for disrupting crystalline domains before the degradation process starts [28], as evidenced by the decreased temperatures corresponding to both the onset of decomposition and the maximum degradation rate in DGT (Fig. 2d and Additional file 1: Table S1). The lower decomposition temperatures after ball milling also indicate that ball-milled corn stover has a lower activation energy, enabling it easier to transform under catalytic conditions. This was demonstrated in a number of studies where ball milling pretreatment was shown to promote the catalytic conversion of lignocellulosic biomass [21, 24, 25].

To further explore the reactivity of corn stover, the changes in inter- and intra-molecular linkages were evaluated by quantifying the available reducing ends, which are highly reactive aldehyde carbonyl groups at one end of polysaccharide molecules [29]. Figure 2e shows that the reducing-end concentration of corn stover increases continuously with increased ball milling time, indicating that more available reducing ends emerge during the ball milling process. Such increase of reducing ends induced by ball milling has been intensively investigated [15, 16, 21]. The increased number of reducing ends implies that ball milling breaks the β-1,4-glycosidic bonds in cellulose chains along with the significant reduction in particle size. The breakage of glycosidic bonds to form more active reaction sites can consequently improve the corn stover degradability.

### Behaviors of varisized corn stover in enzymatic saccharification and acid-catalyzed alcoholysis

**Fig. 3:**
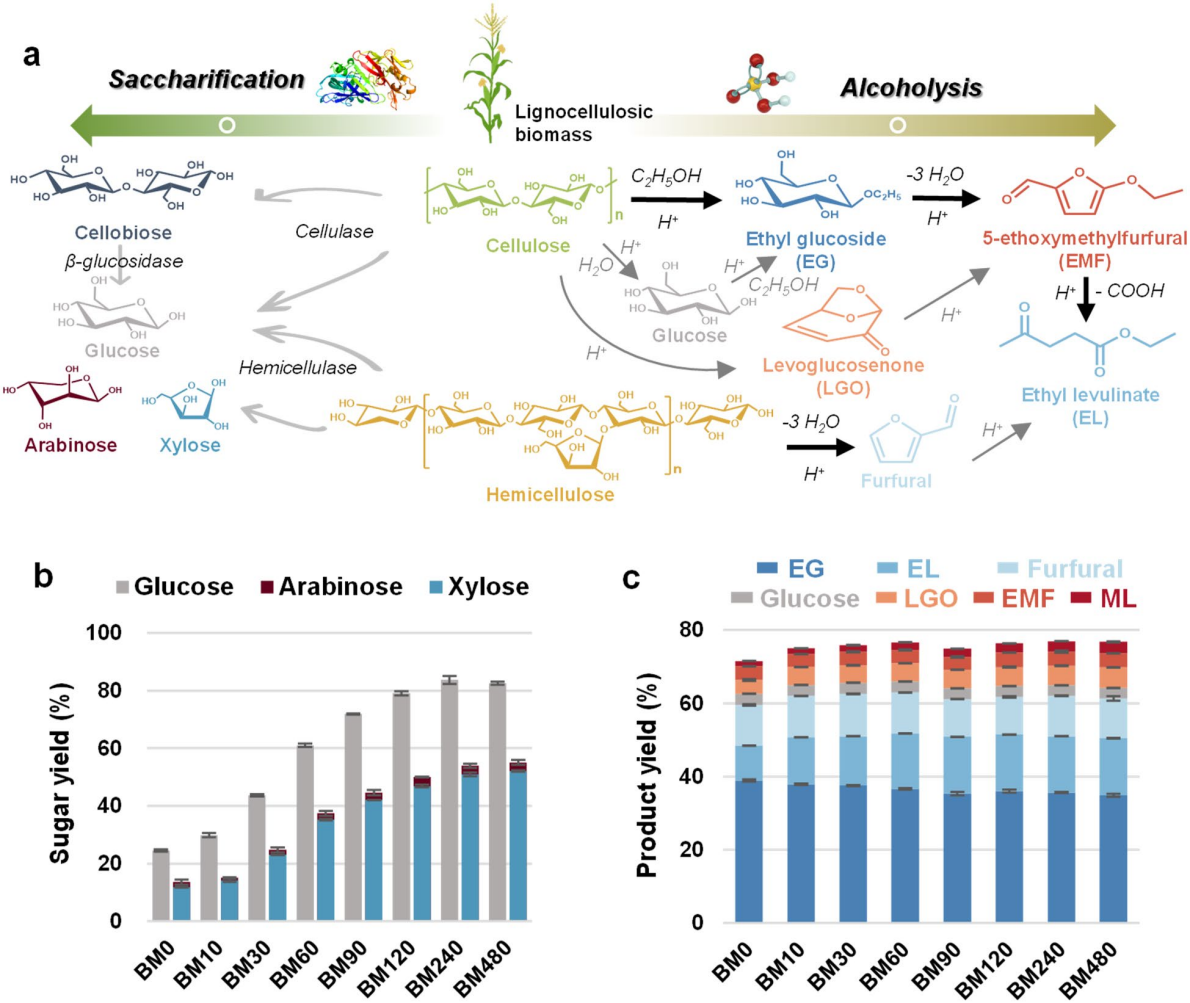
Conversion of varisized corn stover. **a** Reaction routes. **b** Enzymatic saccharification. Reaction conditions: 50 mg of corn stover samples, 5 FPU g^−1^ solids of enzyme (Cellic® Ctec2), 1 mL of 50 mM citrate buffer (pH 4.8), 50 °C, 72 h. **c** Acid-catalyzed alcoholysis. Reaction conditions: 1 g of corn stover samples, 0.2 g of H_2_SO_4_, 20 g of ethanol, 160 °C, 30 min. Error bars indicate standard deviation from duplicate measurements

The chemical and structural properties of lignocellulosic biomass are largely responsible for its susceptibility to enzymatic or acidic hydrolysis [12], thus the changes in these properties as a result of ball milling affect the lignocellulose conversion. Figure 3b describes the effect of ball milling on corn stover digestibility, at 5% solids loading enzymatic hydrolysis, using 5 FPU cellulase per g solids enzyme loading. The results suggest that ball milling is highly efficient for improving the saccharification of corn stover. Enzymatic hydrolysis of untreated corn stover (BM0) for 72 h only results in 24.6% glucan and 11.82% xylan to be saccharified to glucose and xylose, respectively. The saccharification is substantially enhanced for corn stover after ball milling, releasing > 60% glucose and > 35% xylose after ball milling for greater than 60 min. The results coincide with previous results in which ball milling pretreatment was found to notably enhance the sugar release from various agricultural residues [15, 23]. This enhancement could be explained by the fact that ball milling efficiently alters the physicochemical properties of corn stover. As a result, the β-1,4 glucosidic bonds in cellulose are more accessible and readily hydrolyzed by cellulase, generating cellobiose and glucose, and the further breakdown of cellobiose into glucose is accomplished by the β-glucosidase [30]. Meanwhile, the digestion of hemicellulose by hemicellulase is also improved to more release xylose and arabinose (Fig. 3a).

Apart from enzymatic saccharification, the alcoholysis of corn stover by sulfuric acid to ethyl levulinate (EL) is also improved through ball milling. As depicted in Fig. 3c, ball milling the corn stover for 60 min increases the EL yield from 9.47% to 15.13%, consistent with our previous study that ball milling facilitates the transformation of corn stover to EL [21, 31]. However, further extending the ball milling time does not result in significant improvement of EL yield, implying that the susceptibility of ball-milled corn stover to acid-catalyzed alcoholysis differs from that to enzymatic saccharification. As illustrated in Fig. 3a, acid-catalyzed alcoholysis of corn stover to EL involves several steps [31]. First, the low molecular weight fragments are produced from the cellulose polymer chains by the action of acid catalysts and end up as ethyl glucoside (EG, including ethyl-α-d-glucopyranoside and ethyl-β-d-glucopyranoside) dissolved in the ethanol medium. The EG decomposes to 5-ethoxymethyfurfural (EMF), which is further transformed in a serial mode to EL as the end product. All anticipated products were detected and analyzed using GC and HPLC analyses. In addition to the anticipated products, small amounts of intermediates (e.g., glucose and levoglucosenone) and substantial furfural were observed in the reaction mixture. The generation of glucose is the result of cellulose breakdown in the presence of water, and it can directly react with ethanol to form EG. Levoglucosenone (LGO) is regarded as a cellulose decomposition product which on subsequent dehydration can yield EMF [32, 33]. The formation of furfural is attributed to the presence of hemicellulose, which may produce xylose followed by dehydration to furfural and its conversion to EL through a series of reactions [31, 32]. During all experiments, methyl levulinate (ML) was also observed in the reaction mixture. It is a known product of the acid-catalyzed alcoholysis of biomass carbohydrates in methanol-rich medium [8]. It is likely that methanol was generated during the alcoholysis of corn stover, producing ML [31].

### Relationships between physicochemical properties and degradability

**Fig. 4:**
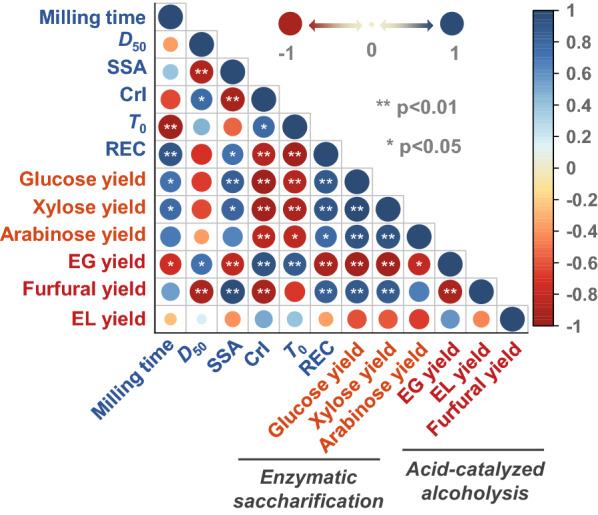
Matrix of Pearson correlation coefficients among physicochemical properties and main product yields. * indicates a significant correlation between parameters (*p* < 0.05). ** indicates an extremely significant correlation between parameters (*p* < 0.01)

As mentioned above, the chemical and structural properties of corn stover change drastically with ball milling, and all of these changes could contribute to its enhanced digestibility. Considering the potential interactions among these physicochemical parameters, Pearson correlation analysis was performed to study the correlations between various physicochemical characteristics and enzymatic saccharification as well as acid-catalyzed alcoholysis (Fig. 4). Ball milling pretreatment converts macroscopic particles of corn stover into smaller and more reactive fragments, with concomitant changes in the physicochemical factors, including specific surface area, crystallinity, thermal stability, and reducing-end concentration. These factors are herein strongly correlated to glucose and xylose yields (Fig. 4), consistent with previous report that increased special surface area, decreased crystallinity, and breakage of linkages in polysaccharides significantly improved enzymatic digestibility [15]. In contrast, these factors are not significantly correlated to EL yield, although they exhibit an extremely significant correlation with EG yield (*p* < 0.01). This difference is likely due to the complexity of alcoholysis process, making the final product EL less susceptive to the substrate-related factors than the intermediate EG. Unexpectedly, not only EL yield but also glucose and xylose yields seem to have no significant correlation with particle size, which is in sharp contrast to the change in properties and increase in digestibility when particle size is reduced by ball milling. Identical conclusions were obtained in past studies that particle size has a limited impact on biomass enzyme digestibility [34].

**Fig. 5:**
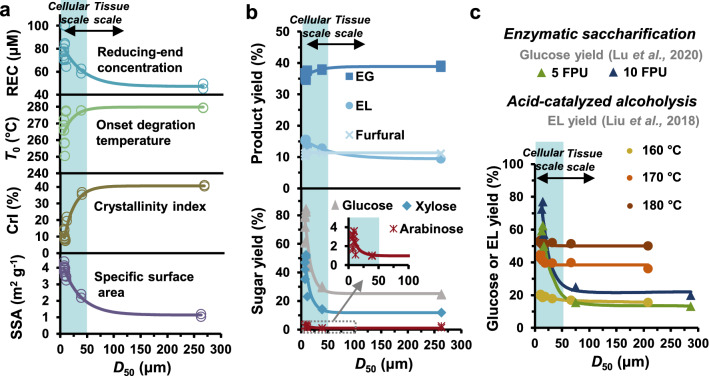
Physicochemical properties and digestibility of corn stover as a function of particle size (*D*_50_). **a** Physicochemical properties; **b** enzymatic saccharification (top) and acid-catalyzed alcoholysis (bottom) of corn stover as a function of *D*_50_ as well as **c** previously reported results under varying reaction conditions. Results collected from publications for both enzymatic saccharification (glucose yield) and catalytic alcoholysis (EL yield) of varisized corn stover. Added trend lines are used to indicate changing trends. Data taken from Lu et al. [20] (enzymatic saccharification) and Liu et al. [21] (acid-catalyzed alcoholysis)

To gain more insights into the effect of size reduction on biomass properties as well as subsequent conversion, the relationships among structure, properties, and digestibility of corn stover were further analyzed using the data reported here and previously [20, 21]. As seen in Fig. 5a, all of the mentioned substrate-related properties do not show significant changes at the tissue scale (> 50 μm), while they vary sharply with decreasing particle size when the size reduction reaches cellular scale. It is reasonable that reducing the particle size into a cellular scale rather than only into a tissue scale enables significant changes in surface morphology, crystallinity, thermal stability, and polysaccharide linkages due to the destruction of cell lumen, coinciding with the results of previous studies [15, 17, 18]. As a consequence, corn stover exhibits significant enhancements in digestibility at a cellular scale. It can be seen in Fig. 5b that the extent to which size reduction influences enzymatic saccharification is markedly different from that for acid-catalyzed alcoholysis. Smaller particle size is always associated with bigger changes in the former than is the case with the latter. Similar trends have been observed in previous work (Fig. 5c) [20, 21]. Although size reduction is only able to increase product yield of acid-catalyzed alcoholysis to a limited extent, apparent improvements in EL yield are achieved at higher reaction temperatures regardless of biomass particle size. Conversely, enzymatic saccharification of corn stover is not significantly affected by altering process variables that affect the biomass at a cellular scale, whereas it varies to an extent at a tissue scale. Such trends suggest that size reduction to a cellular scale is of greater significance for enzymatic saccharification far beyond other process parameters, which more strongly affect acid-catalyzed alcoholysis.

### Effects of process variables on catalytic conversion

**Fig. 6:**
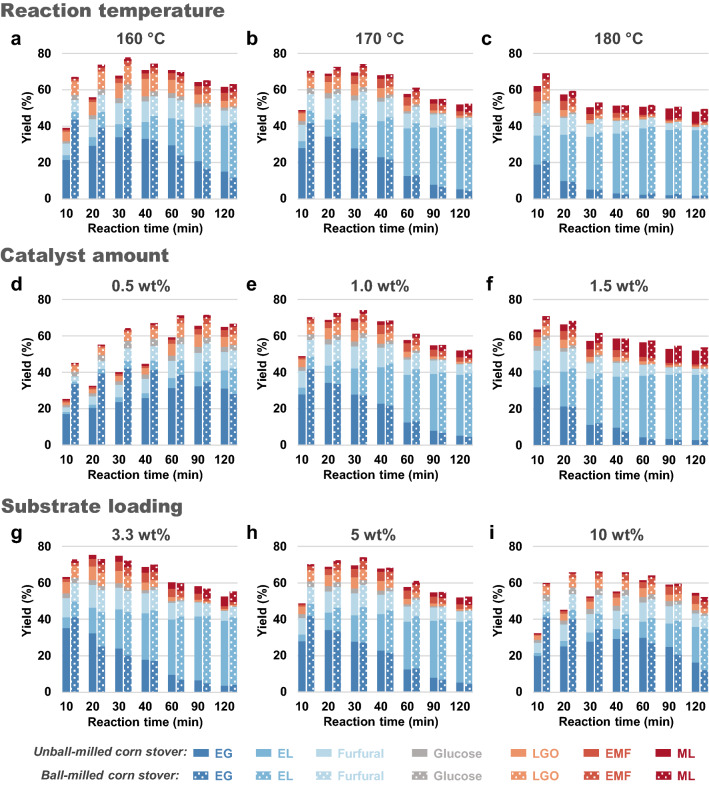
Alcoholysis of untreated and ball-milled corn stover with different **a**–**c** reaction temperatures, **d–f** catalyst amounts, and **g**–**i** substrate loadings. Reaction conditions: **a**–**c** 1 g of substrate (5 wt% of solvent amount), 0.2 g of H_2_SO_4_ (1.0 wt% of solvent amount), 20 g of ethanol; **d**–**f** 1 g of substrate (5 wt% of solvent amount), 20 g of ethanol, 170 °C; **g**–**i** 0.2 g of H_2_SO_4_ (1.0 wt% of solvent amount), 20 g of ethanol, 170 °C

The impact of process variables, including reaction temperature, catalyst amount, substrate loading, and reaction time, on the alcoholysis of corn stover was then systematically investigated. Figure 6 depicts the product distributions of both untreated and ball-milled corn stover under a wide range of reaction conditions. Among the tested variables, reaction temperature seems to be the most critical factor impacting the product distributions of alcoholysis. At 160 °C, corn stover samples after and before ball milling up to 60 min yield only 19.62 and 15.00% of EL, respectively, leaving a large proportion of EG in the mixture (Fig. 6a). Significant improvements in EG conversion are observed at higher temperatures, culminating with > 36% EL yield for both untreated and ball-milled corn stover after 60 min at 180 °C (Fig. 6c). It should be noted that higher temperatures reduce or eliminate the benefits from ball milling with minor differences in product distribution of samples before and after ball milling. A similar trend is observed for the effects of catalyst amount. The yield of EL is substantially improved when applying higher sulfuric acid concentrations, while the difference in product distributions before and after ball milling is correspondingly reduced. The product distribution is also clearly function of the substrate loading, with high substrate loadings accompanied by reduced EL yield. This observation suggests that side reactions of corn stover to EL are likely second or higher-order and more competitive with the reaction to EL at higher substrate concentrations, resulting in lower EL yield.

In all cases, the product distributions of ball-milled corn stover differ markedly from those of unmilled corn stover at the initial stage of reaction. However, this difference narrows with prolonged reaction time, implying that ball milling tends to result in favoring alcoholysis at shorter reaction times. As anticipated on the basis of Fig. 3a, the yields of both EG and EMF have a maximum. The maximum EMF yields obtained in all experiments are generally much lower than the maximum EG yields, indicating that the transformation of EMF to EL is much faster than its formation from EG.

**Fig. 7:**
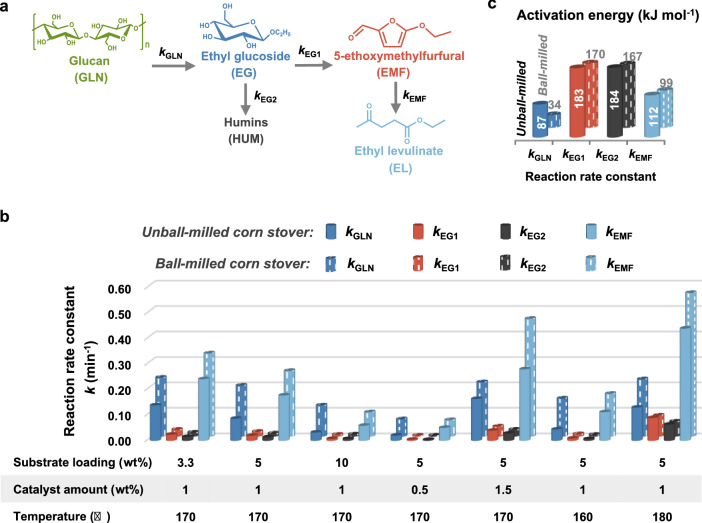
Kinetic study on the alcoholysis of untreated and ball-milled corn stover. **a** Simplified reaction model for kinetic study. **b** Rate constants (*k*) for the alcoholysis of untreated and ball-milled corn stover under various reaction conditions and corresponding **c** activation energies. Reaction conditions: 20 g of ethanol, substrate loading and catalyst amount added on the basis of solvent

To further elaborate the effects of ball milling and process parameters, a kinetic study on the alcoholysis of both untreated and ball-milled corn stover was conducted. A simplified kinetic model was used to evaluate the kinetics of corn stover alcoholysis considering the complex reaction pathways and unstable intermediates (Fig. 7a, see Additional file 1 for details) [21]. As can be seen from Fig. 7b, the rate constants of both corn stover decomposition (*k*_GLN_) and EL generation (*k*_EMF_) increase after ball milling, confirming the positive effect of ball milling on EL formation. Similarly, reducing substrate loading, increasing catalyst amount, or raising reaction temperature also favors the production of EL. However, the rate constants of undesired products such as humins also increase, which is particularly evident when the temperature rises. Therefore, a mild reaction temperature is favored for highly selective conversion of corn stover. It is noticeable that the rate constant of corn stover decomposition is far less than that of EL generation for untreated corn stover, while ball-milled corn stover exhibits a significantly enhanced rate constant of its decomposition, which is even comparable to that of EL generation under some mild conditions. These observations not only indicate that the decomposition of corn stover is a rate-determining step in alcoholysis, but also demonstrate the profound effect of ball milling on this key step, especially under mild reaction conditions. The activation energy values in Fig. 7c, derived from the Arrhenius plot (Additional file 1: Fig. S4), show that ball milling prominently reduces the activation energy of corn stover decomposition but has a negligible impact on other steps. This result is in good agreement with the correlation analysis that particle size reduction has a more prominent influence on EG yield relative to EL yield.

**Fig. 8:**
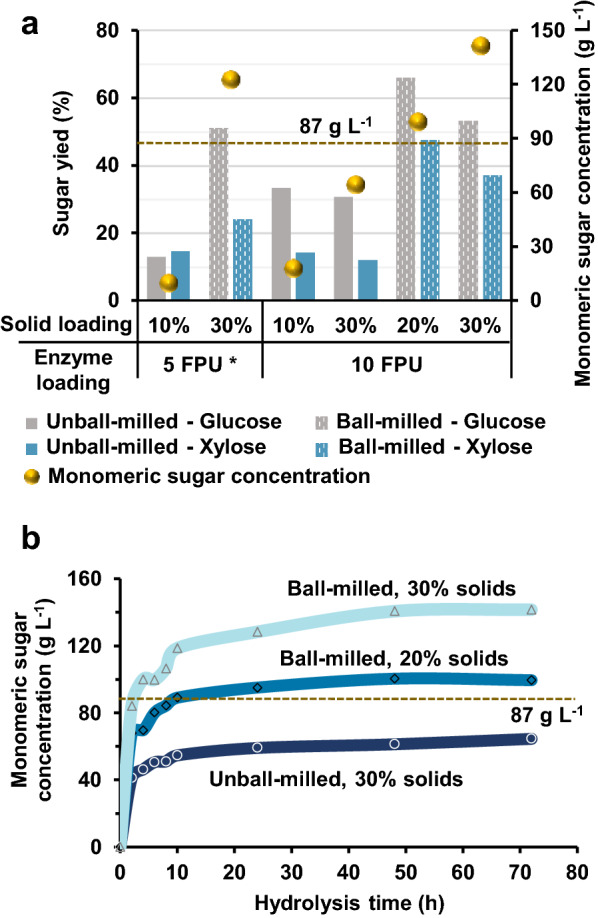
Enzymatic hydrolysis studies for untreated and ball-milled corn stover at various process conditions. **a** Effect of solid loading and enzyme loading on 72 h glucose and xylose yield and their combined concentration in the reaction mixture. **b** Typical profiles for monomeric sugar concentration as a function of hydrolysis time at 10 FPU g^−1^ solids with different conditions. The horizontal-dashed line indicates the monomeric sugar concentration threshold (87 g L^−1^) above which the distillation of ethanol is cost-effective. * Data taken from Lu et al. [20]

To better understand the potential benefits of size reduction on enzymatic saccharification, it is necessary to evaluate the ball-milled feedstocks under more industrially relevant conditions. Herein, both untreated and ball-milled corn stover were subjected to enzymatic hydrolysis at 10%, 20%, and 30% solids loading for 72 h at 5 FPU enzyme per g solids and 10 FPU enzyme per g solids. Figure 8a compares the monomeric sugar release from untreated and ball-milled corn stover under various enzymatic hydrolysis conditions. Ball-milled corn stover is more digestible relative to untreated corn stover for all tested conditions. However, the benefits of ball milling become more evident at lower enzyme loadings, implying that the ball-milled corn stover exhibits high digestibility even under enzyme-limiting conditions. Higher sugar yields after 72 h are observed for ball-milled corn stover at 5 FPU g^−1^ solids and 30% solids loading compared to hydrolysis of untreated corn stover at 10 FPU g^−1^ solids and the same or even lower solids loading. This observation demonstrates that the ball milling process can achieve considerable fermentable sugar yield at high solids and low enzyme loading, which are the two most crucial factors responsible for the competitiveness of enzymatic saccharification [6, 35]. Additionally, at either 20% or 30% solids loading, the total released monomeric sugar concentration of ball-milled corn stover exceeds 87 g L^−1^, which is the minimum fermentable sugar concentration for cost-effective ethanol distillation in the subsequent process [20, 36]. Moreover, this target concentration can be reached within 12 h (Fig. 8b), revealing the high efficiency of enzymatic hydrolysis of ball-milled corn stover. Meanwhile, ball-milled corn stover has been reported to have reduced slurry viscosity [20]. The improved rheological behavior is also central to industrial use by alleviating the mixing problems of high solids slurries, thus eventually driving down the capital and production costs [6]. This is supported by previous work showing that the reduced mixing energy could offset the energy required for ball milling [20].

## Conclusions

Here we show that significant changes in structure and properties along with a size reduction to cellular scale can be achieved by ball milling, resulting in diverse impacts on subsequent degradation of corn stover by enzymes or acid-catalyzed alcoholysis. Detailed characterization of varisized corn stover samples revealed that their chemical and structural features rarely vary at tissue scale, while exhibiting dramatic changes at cellular scale, contributing to significant improvements in enzymatic hydrolysis. In contrast, these parameters are relatively minor contributors to acid-catalyzed alcoholysis, which is more dependent on reaction conditions. Additionally, despite highly relying on process variables, alcoholysis of ball-milled corn stover could significantly reduce the energy barrier of corn stover decomposition compared to using untreated corn stover. Since milder conditions result in more favorable yields from the reactions occurring in the solvent phase, more efficient cellulose and hemicellulose depolymerization at mild conditions for ball-milled corn stover is desirable for industrial application. Furthermore, ball-milled corn stover generates considerable fermentable sugars via enzymatic hydrolysis, with a concentration higher than the threshold necessary for cost-effective ethanol distillation, at a low enzyme loading of 5 FPU g^−1^ solids and 30% solid loading enzymatic hydrolysis. Understanding these fundamentals helps us optimize the lignocellulose preparation process especially for size reduction and provides the guidance for high-solids enzymatic hydrolysis and efficient acid-catalyzed alcoholysis under mild conditions.

## Methods

### Materials

Corn stover was obtained from farmland (Qujing, Yunnan, China). The main composition of the corn stover (on a dry weight basis) was 33.20% cellulose, 21.23% hemicellulose, 22.84% lignin, and 6.84% ash, determined using the NREL/TP-510-42618 method [37]. Methyl levulinate (ML), ethyl levulinate (EL), levoglucosenone (LGO), furfural, ethyl α-d-glucopyranoside (α-EG) and ethyl β-d-glucopyranoside (β-EG), henceforth, “EG”, together, were supplied by TCI (Shanghai, China). Glucose and 5-ethoxymethylfurfural (EMF) were all of the analytical grades purchased from Sigma-Aldrich (Shanghai, China). Ethanol and sulfuric acid were bought from the Beijing Chemical Works (Beijing, China). Ultrapure water (Milli-Q, Millipore, Billerica, MA, USA) was used for solution preparation.

### Preparation of ball-milled corn stover samples

After air-drying, the raw corn stover was manually cut into 3–5 cm segments and then coarsely ground through a 1.00-mm screen by an RT-34 milling machine (Hongquan Pharmaceutical Machinery Ltd., Hong Kong, China). The resulting corn stover sample, denoted as BM0, had a moisture content of 4%. Then, a CJM-SY-B ultrafine vibration ball mill (Qinhuangdao Taiji Ring Nano-Products Co., Ltd., Hebei, China) was applied to produce the ball-milled corn stover samples. The BM0 was placed in a zirconia tank (2 L) milled with zirconia balls (6–10 mm in diameter) at a volume ratio of 1:2 for 10, 30, 60, 90, 120, 240, and 480 min, respectively, the resulting samples were denoted as BM10, BM30, BM60, BM90, BM120, BM240, and BM480, respectively. During the ball milling process, the instrument was equipped with a cooling circulating water system to control the temperature below 30 °C.

### Characterization of corn stover in the ball milling process

#### Field emission scanning electron microscopy analysis

The surface morphologies of corn stover samples were analyzed by a JSM-6700 field emission scanning electron microscope (FE-SEM, JEOL Ltd., Tokyo, Japan) at an accelerating voltage of 10 kV. The samples were sprayed with a fine layer of gold using an ion sputter coater before the examination.

#### Particle size measurements

The particle size distribution of corn stover samples was determined from 0.01 to 3000 μm by the Mastersizer 3000 laser diffraction particle analyzer (Malvern Instruments Ltd., Worcestershire, United Kingdom) with the Aero S accessory. An assumption that all particles are spherical was applied to calculate the *D*_10_, *D*_50,_ and *D*_90_ from the curves of particle size distribution, which represent the 10th, 50th and 90th percentiles of the total volume, respectively. The median particle size (*D*_50_) was used to characterize the particle size distribution, and the overall heterogeneity of the particle size was evaluated by the span, defined as (*D*_90_–*D*_10_)/*D*_50_. All samples were measured in duplicate.

#### Specific surface area analysis

A multipurpose Micromeritics Tristar II apparatus (Micromeritics Instrument Corp., Georgia, USA) was used to measure the specific surface area (SSA) of corn stover at the boiling temperature of liquid nitrogen after a 4-h degassing at 105 °C. The SSA was obtained according to the Brunauer–Emmett–Teller (BET) method [38].

### X-ray diffraction analysis

X-ray diffraction (XRD) patterns were obtained from the XD-3 X-ray diffractometer (Beijing Purkinje General Instrument Co., Ltd., Beijing, China) with Cu-Kα at 40 kV and 30 mA. The scanning range of 2*θ* was between 5° and 40° with a step size of 0.2° and a rate of 2° min^−1^. The crystallinity index (CrI) was determined using an empirical approach [39] based on the XRD curves:

$$CrI \left( \% \right) = \frac{{I_{002} - I_{am} }}{{I_{002} }} \times 100,$$

where *I*_*002*_ is the maximum intensity of the (002) peak, *I*_*am*_ is the intensity of the amorphous region at approximately 2*θ* = 18°.

#### Thermogravimetric analysis (TGA)

Pyrolysis tests of corn stover samples were performed using an SDT Q600 Thermogravimetric Analyzer (TA Instruments, Inc., New Castle, Pennsylvania, USA) in a nitrogen atmosphere. Approximately 5 mg samples were taken in standard aluminum pans and an empty pan was used as a reference. Thermograms were obtained by heating the samples from ambient temperature to 1000 °C at a heating rate of 20 °C min^−1^ under a nitrogen flow of 100 cm^3^ min^−1^. The weight-loss rate was obtained from the first derivative of thermogravimetric (DTG) data.

#### Reducing-end determination

The molar reducing-end concentration (REC) of the corn stover was determined by a modified BCA method previously described [40, 41]. Briefly, 5 mL of BCA working solution was mixed with 2 mg of sample dispersed in 5 mL of water in a tube with stopper. The BCA working solution was prepared fresh by mixing equal volumes of solution A containing 0.971 g of disodium 2,2'-bicinchoninate, 27.14 g of Na_2_CO_3_, and 12.1 g of NaHCO_3_ dissolved in 500 mL of distilled water, with solution B containing 0.624 g of CuSO_4_∙5H_2_O and 0.631 g of ʟ-serine dissolved in 500 mL of water. After an incubation at 75 °C for 30 min, the tubes were cooled to room temperature immediately. Then, the reaction mixture was transferred into a centrifuge tube and centrifuged for 5 min to remove the solids. The absorbance of supernatant was measured at 560 nm. The concentration of reducing end was determined by comparison with a calibration curve prepared using glucose solutions with concentrations ranging 0–100 μM.

### Catalytic conversion of corn stover

#### Enzymatic saccharification experiment

Enzymatic saccharification of different corn stover samples was carried out in 50 mM citrate buffer (pH 4.8) at a solid loading of 5% (w/v) in an incubator shaker (50 °C, 150 rpm). Commercial cellulase (Cellic CTec2, Novozymes) was loaded at 5 FPU g^−1^ solids. The microbial interference was controlled by the addition of tetracycline hydrochloride (0.08 g L^−1^). After digestion for 72 h, the resulting mixture was transferred in a boiling water bath for 10 min to inactive the cellulase. Then, the released sugars were analyzed using an HPLC system (Hitachi L-7200, Hitachi, Tokyo, Japan) equipped with a refractive index detector and a Bio-Rad Aminex HPX-87P column at 80 °C. Ultrapure water was used as eluent at a flow rate of 0.6 mL min^−1^. Each experiment was performed in duplicate.

#### Microwave-assisted alcoholysis procedure

The alcoholysis of corn stover samples was conducted using an ETHOS UP microwave system (Milestone Srl, Sorisole, BG, Italy). One g of corn stover was added into a 100-mL Teflon reaction vessel with 0.2 g H_2_SO_4_ and 20 g ethanol. The mixture was heated up to 170 °C within 2 min and then kept at this temperature for 30 min with magnetic stirring. After the reaction, the vessel was cooled in an ice-water bath to ambient temperature before collecting the mixture. The resulting mixture was sampled (1 g) and dissolved in methanol or water, passing through a 0.22 μm PTFE membrane filter before analysis. All experiments were carried out in duplicate.

The reaction products and intermediates (EL, ML, and LGO) were quantitatively analyzed by a Shimadzu GC-2014C gas chromatograph (GC, Shimadzu Corp., Kyoto, Japan) using a flame ionization detector (FID) fitted with a DB-5 capillary column (30 m × 0.25 mm × 0.25 μm). The carrier gas was nitrogen with a flow rate of 1.0 mL min^−1^. The injection port and detector temperature were set at 220 °C and 250 °C, respectively. The oven temperature was programmed as previously described [8]. Analysis of other products, glucose, EG, EMF, and furfural, was performed by using a high-performance liquid chromatography (HPLC, Waters e6295, Waltham, MA, USA) equipped with an Aminex HPX-87H column (300 mm × 7.8 mm, Bio-Rad) at 55 °C and a refractive index detector (Waters 2414) at 50 °C. The eluent was 5 mM sulfuric acid solution at a flow rate of 0.6 mL min^−1^.

The concentration of compounds in the reaction mixture was quantified by comparison with calibration curves from the standard solutions of known concentrations. As the target product EL can be produced from both cellulose and hemicellulose [31], the product yield from corn stover was calculated on a molar basis of total carbohydrates below:

$${\text{Product yield }}\left( {{\text{\%}}} \right) = \frac{{\text{Mole of product detected}}}{{\text{Mole of initial monosaccharide unit in corn stover}}}\times100,$$

where the monosaccharide unit includes glucose and xylose, theoretically derived from the glucan and xylan content, respectively.

### Statistical analysis

The statistical analysis of various parameters of corn stover samples was carried out using Duncan’s multiple range test at 95% confidence intervals, and the Pearson correlation coefficient was calculated using the correlation analysis to examine the relationship between the parameters by IBM SPSS Statistics 20.0.

## Supplementary Information

**Additional file 1:** It contains kinetic model, 1 Table, and 4 Figures: Thermal degradation parameters (**Table S1**); TGA decomposition graphs (**Fig. S1**); Correlation matrix of coefficients among all factors (**Fig. S2**); Comparison of experimental data and kinetic model (**Fig. S3**); and Arrhenius plots (**Fig. S4**).

## Data Availability

All data generated or analyzed during this study are included in this published article.

## Abbreviations

ML: Methyl levulinate
EL: Ethyl levulinate
LGO: Levoglucosenone
EG: Ethyl glucoside
EMF: 5-Ethoxymethylfurfural
SEM: Scanning electron microscopy
SSA: Specific surface area
XRD: X-ray diffraction
CrI: Crystallinity index
TGA: Thermogravimetric analysis
DTG: Differential thermogravimetry
*T*_0_: Onset degradation temperature
REC: Reducing-end concentration
GC: Gas chromatograph
HPLC: High-performance liquid chromatography
