# Supplementary material for: Structure–property–degradability relationships of varisized lignocellulosic biomass induced by ball milling on enzymatic hydrolysis and alcoholysis
Source: Biotechnol Biofuels Bioprod. 2022 Apr 4;15:36. doi: 10.1186/s13068-022-02133-x (PMC8981931; doi:10.1186/s13068-022-02133-x)
Supplement: Supplementary file 1 — Additional file 1: It contains kinetic model, 1 Table, and 4 Figures: Thermal degradation parameters (Table S1); TGA decomposition graphs (Fig. S1); Correlation matrix of coefficients among all factors (Fig. S2); Comparison of experimental data and kinetic model (Fig. S3); and Arrhenius plots (Fig. S4). [file 13068_2022_2133_MOESM1_ESM.docx]

**Structure-property-degradability relationships of varisized lignocellulosic biomass induced by ball milling on enzymatic hydrolysis and alcoholysis**

Xueli Chen ^a, b, c^, Dingping He ^a^, Tao Hou ^a^, Minsheng Lu ^d^, Nathan S. Mosier ^b, c^, Lujia Han ^a^, Weihua Xiao ^a, *^

*^a^ Engineering Laboratory for AgroBiomass Recycling & Valorizing, College of Engineering, China Agricultural University, Beijing 100083, China*

*^b^* *Laboratory of Renewable Resources Engineering (LORRE), Purdue University, West Lafayette, IN 47907, United States*

*^c^ Department of Agricultural and Biological Engineering, Purdue University, West Lafayette, IN 47907, United States*

*^d^ School of Light Industry and Food Engineering, Guangxi Key Laboratory of Clean Pulp & Papermaking and Pollution Control, Guangxi University, Nanning 530004, China*

^*^ Corresponding author: Dr. Weihua Xiao,

P.O. Box 191, College of Engineering,

China Agricultural University (East campus),

17 Qing-Hua-Dong-Lu, Haidian district,

Beijing 100083, P. R. China.

Telephone: 86-10-62736778, Fax: 86-10-62736778

Email: xwhddd@163.com

# Kinetic model

Acid-catalyzed alcoholysis of corn stover is a complex process involving a variety of steps and products (Fig. 3a), analogous to the acidic hydrolysis in water [[1](#_ENREF_1), [2](#_ENREF_2)]. Considering the process complexity, several assumptions were made to simplify the reaction model based on previous studies [[1](#_ENREF_1), [2](#_ENREF_2)] and experimental results. (1) Only the glucan component was considered as the source for ethyl levulinate (EL) production because not much EL is generated from xylan [[3](#_ENREF_3)]. (2) The first step in acid-catalyzed alcoholysis of glucan (GLN) was the cleavage of the β-1,4-glycosidic bonds to form ethyl glucoside (EG) while the formation of glucose is neglected because of its low content (Fig. 6). (3) The desired outcome of EG decomposition is the generation of 5-ethoxymethylfurfural (EMF), but it is also accompanied by the formation of humins and other undesirable products, which are considered humins (HUM) for simplicity. (4) EL is defined as the subsequent degradation product of EMF and the decomposition of EMF to humins is not included since the reaction rate for EMF conversion is relatively high, which is evidenced by the always low concentration of EMF during the alcoholysis reaction due to its conversion is much faster than its formation. (5) The final product EL is table at the applied conditions as there is no significant decrease by prolonging reaction time in the wide range of reaction conditions employed (Fig. 6). Based on these assumptions, a pseudo-first-order reaction model on the production of EL from corn stover is proposed as depicted in Fig. 7a.

This reaction model results in the following first-order differential rate equations:

$\frac{d[GLN]}{dt}=-k_{GLN}[GLN]$ (1)

$\frac{d[EG]}{dt}=k_{GLN}[GLN]-k_{EG1}[EG1]-k_{EG2}[EG2]$ (2)

$\frac{d[EMF]}{dt}=k_{GLN}[EG1]-k_{EMF}[EMF]$ (3)

$\frac{d[EL]}{dt}=k_{EMF}[EL]$ (4)

Solving the linear differential rate equations above, the time-dependent expressions are obtained below.

$\left[ GLN \right]={[GLN]}_{0}exp(-k_{GLN}t)$ (5)

$[\mathrm{EG}]=\frac{k_{GLN}{[GLN]}_{0}}{k_{EG1}+k_{EG2}-k_{GLN}}[\exp\left( -k_{GLN}x \right)-\exp\left( -(k_{EG1}+k_{EG2})x \right)]$ (6)

$\left[ EMF \right]=\frac{k_{GLN}k_{EG1}\left[ GLN \right]_{0}}{k_{EG1}+k_{EG2}-k_{GLN}}\left[ \frac{\exp\left( -k_{GLN}x \right)}{k_{EMF}-k_{GLN}}-\frac{\exp\left( -\left( k_{EG1}+k_{EG2} \right)x \right)}{k_{EMF}-\left( k_{EG1}+k_{EG2} \right)} \right]+ \frac{k_{GLN}k_{EG1}\left[ GLN \right]_{0}\exp\left( -k_{EMF}x \right)}{\left( k_{EMF}-k_{GLN} \right)k_{EMF}-\left( k_{EG1}+k_{EG2} \right)}$(7)

$\left[ EL \right]=\frac{k_{GLN}k_{EG1}k_{EMF}\left[ GLN \right]_{0}}{k_{EG1}+k_{EG2}-k_{GLN}}\left[ \frac{\exp\left( -k_{GLN}x \right)-1}{k_{GLN}\left( k_{GLN}-k_{EMF} \right)}-\frac{\exp\left( -\left( k_{EG1}+k_{EG2} \right)x \right)-1}{\left( k_{EG1}+k_{EG2} \right)\left( k_{EG1}+k_{EG2}-k_{EMF} \right)} \right]- \frac{k_{GLN}k_{EG1}\left[ GLN \right]_{0}\left( \exp\left( -k_{EMF}x \right)-1 \right)}{\left( k_{GLN}-k_{EMF} \right)\left( \left( k_{EG1}+k_{EG2} \right)-k_{EMF} \right)}$(8)

where [GLN], [EG], [EMF] and [EL] represent the yield of glucan, EG, EMF and EL at time t (min), in mol%, while [GLN]_0_ is the initial content of glucan (100 mol%). *k*_GLN_ (min^-1^), *k*_EG1_ (min^-1^), *k*_EG2_ (min^-1^), and *k*_EMF_ (min^-1^) are reaction rate constants (min^-1^).

The kinetic parameters in the equations were fitted to simulate the yields of glucan residue, EG, EMF, and ML obtained under tested conditions, respectively, using the method of non-linear least squares regression analyses by MATLAB.

Fig. S3 shows a good fit between the experimental yield of MG and ML and the kinetic model under different conditions.

**Table S1.** Onset degradation temperature (*T*_0_), maximum thermal degradation temperature (*T*_max_) and maximum weight loss rate (*R*_max_) in the thermal degradation process of each sample obtained from TG and DTG curves.

| Sample | *T*_0_ (°C) | *T*_max_ (°C) | *R*_max_ (%/°C) |
| --- | --- | --- | --- |
| BM0 | 279.38 ± 0.13^g^ | 323.55 ± 0.18^g^ | 18.44 ± 0.3^f^ |
| BM10 | 277.82 ± 0.08^f^ | 323.36 ± 0.22^g^ | 17.39 ± 0.08^ef^ |
| BM30 | 277.44 ± 0.38^f^ | 321.27 ± 0.07^f^ | 17.16 ± 0.02^de^ |
| BM60 | 272.33 ± 0.08^e^ | 318.63 ± 0.25^e^ | 17.58 ± 0^cd^ |
| BM90 | 268.98 ± 0.1^d^ | 317.19 ± 0.05^d^ | 16.9 ± 0.08^c^ |
| BM120 | 265.79 ± 0.13^c^ | 316.04 ± 0.1^c^ | 16.33 ± 0.11^c^ |
| BM240 | 258.17 ± 0.19^b^ | 314.65 ± 0.22^a^ | 14.34 ± 0.01^b^ |
| BM480 | 250.48 ± 0.05^a^ | 315.34 ± 0.19^b^ | 13.28 ± 0.46^a^ |

Data are presented as mean ± standard deviation. Values in the same column with different letters are significantly different (p < 0.05).

**Fig. S1.** TGA decomposition graphs showing relative sample weight of different corn stover samples.

**Fig. S2.** Correlation matrix of coefficients among all factors.

**Fig. S3.** Comparison of experimental data (▲：EG, ×: EMF, ●: EL) and kinetic model (solid lines). Reaction condition: 20 g of ethanol, substrate loading and catalyst amount added on the basis of solvent.

**Fig. S4.** Arrhenius plot in the range from 160 °C to 180 °C for the alcoholysis of (a) untreated corn stover and (b) ball-milled corn stover.

# References

1. Girisuta B, Dussan K, Haverty D, Leahy JJ, Hayes MHB: **A kinetic study of acid catalysed hydrolysis of sugar cane bagasse to levulinic acid**. *Chem Eng J* 2013, **217**:61-70.

2. Zheng X, Zhi Z, Gu X, Li X, Rui Z, Lu X: **Kinetic study of levulinic acid production from corn stalk at mild temperature using FeCl_3_ as catalyst**. *Fuel* 2017, **187**:261-267.

3. Liu H, Chen X, Zhang Y, Lu M, Lyu H, Han L, Xiao W: **Alcoholysis of Ball-Milled Corn Stover: The Enhanced Conversion of Carbohydrates into Biobased Chemicals over Combination Catalysts of [Bmim-SO_3_H][HSO_4_] and Al_2_(SO_4_)_3_**. *Energy & Fuels* 2020, **34**(6):7085-7093.
